# Expression of PPAR*δ* in multistage carcinogenesis of the colorectum: implications of malignant cancer morphology

**DOI:** 10.1038/sj.bjc.6603343

**Published:** 2006-09-12

**Authors:** O Takayama, H Yamamoto, B Damdinsuren, Y Sugita, C Y Ngan, X Xu, T Tsujino, I Takemasa, M Ikeda, M Sekimoto, N Matsuura, M Monden

**Affiliations:** 1 Department of Surgery, Gastroenterological Surgery, Graduate School of Medicine, Osaka University, 2-2 Yamada-oka, Suita-City, Osaka 565-0871, Japan; 2 Department of Pathology, School of Allied Health Science, Faculty of Medicine, Osaka University; Osaka 565-0871, Japan

**Keywords:** PPAR*δ*, colorectal cancer, malignant morphology, *β*-catenin

## Abstract

Whether peroxisome proliferator-activated receptor (PPAR) *δ* is a good target for the chemoprevention and/or treatment of colorectal cancer (CRC) remains controversial. Our goal was to examine PPAR*δ* expression in multistage carcinogenesis of the colorectum and to assess the relevance of PPAR*δ* in CRC. Immunohistochemical analysis indicated that PPAR*δ* expression increased from normal mucosa to adenomatous polyps to CRC. In cancer tissues, the PPAR*δ* protein was accumulated only in those cancer cells with highly malignant morphology, as represented by a large-sized nucleus, round-shaped nucleus, and presence of clear nucleoli. Interestingly, the cancer tissue often contained both PPAR*δ*-positive and -negative areas, each retaining their respective specific morphological features. Moreover, this pattern persisted even when PPAR*δ*-positive and -negative cells were aligned next to each other within a single cancer nest or gland and was present in the majority of CRC cases. Immunohistochemistry for Ki-67 proliferation marker showed no significant correlation between Ki-67 and PPAR*δ* in CRC samples. Based on Western blot analysis and quantitative RT–PCR, high PPAR*δ* protein expression correlated with high PPAR*δ* mRNA levels. Peroxisome proliferator-activated receptor *δ* may have a supporting role in tumorigenesis, and the close association between PPAR*δ* expression and malignant morphology of CRC cells suggests a pivotal role in cancer tissue.

Peroxisome proliferator-activated receptors (PPARs) are ligand-activated transcription factors belonging to the nuclear hormone receptor superfamily. Peroxisome proliferator-activated receptors play a role in normal physiological processes such as lipid metabolism and embryo implantation, and they have been implicated in the disease-related processes of inflammation, diabetes mellitus, and cancer (Kersten *et al*, 2000; Willson *et al*, 2000). To date, three PPAR isoforms, PPAR*α*, PPAR*δ/β*, and PPAR*γ*, have been isolated (Willson *et al*, 2000). Various functions of the PPAR*α* and *γ* isotypes have been described, such as involvement in lipid homeostasis, immunity, and cellular differentiation (Tontonoz *et al*, 1994; Spiegelman 1998; Kliewer *et al*, 1999). These two isotypes also have clinical significance in the treatment of dyslipidaemia and type II diabetes mellitus (Rangwala and Lazar, 2004). In contrast, less is known about the physiological role of the PPAR*δ* isoform, although there is some evidence supporting its involvement in embryo implantation and development (Lim *et al*, 1999; Barak *et al*, 2002), epidermal maturation and wound healing (Di-Poi *et al*, 2003), and regulation of fatty acid metabolism (Wang *et al*, 2003).

Recent studies suggest that PPAR*δ* may play a role in colorectal cancer (CRC). The adenomatous polyposis coli (*APC*) and *K-ras* genes are known to play a role in colorectal carcinogenesis (Vogelstein *et al*, 1988): PPAR*δ* expression and/or activity increase after loss of the *APC* gene or activation of *K-ras* gene expression (He *et al*, 1999; Shao *et al*, 2002). Cyclooxygenase-2 also modulates intestinal tumorigenesis (Oshima *et al*, 1996), and its metabolite, prostacyclin, increases PPAR*δ* activity in CRC cells (Gupta *et al*, 2000). In addition, PPAR*δ* has also been shown to be a downstream target of APC/*β*-catenin/T-cell factor (TCF)-4-mediated transcriptional activation, which is a key mediator in the development of CRC (He *et al*, 1999). However, it is currently unclear whether PPAR*δ*, like other downstream targets such as c-myc and cyclin D1 (He *et al*, 1998; Tetsu and McCormick, 1999), contributes to oncogenesis and the development of colon tumours. Several studies using Apc^min^ mice, designed to evaluate the role of PPAR*δ* in colon tumour development, have produced conflicting findings. Peroxisome proliferator-activated receptor*δ* was found to be unnecessary for small intestinal polyp formation, but might be required for the development of large-sized intestinal polyps (Barak *et al*, 2002). In addition, PPAR*δ* attenuates polyp formation in chemical and genetic models (Harman *et al*, 2004; Reed *et al*, 2004). In contrast, activation of PPAR*δ* using a synthetic ligand increases the number and size of intestinal polyps (Gupta *et al*, 2004); indeed, PPAR*δ*-deficient CRC cells can establish tumours when grown as xenografts in nude mice (Park *et al*, 2001). In this study, we examined expression of PPAR*δ* in multistage carcinogenesis of the colorectum in an effort to elucidate the role of PPAR*δ* in human CRC.

## MATERIALS AND METHODS

### Cell lines

The IEC18 intestinal cell line was a generous gift from Dr I Bernard Weinstein (Herbert Irving Comprehensive Cancer Center, College of Physicians and Surgeons, Columbia University, New York, NY, USA). They were grown in Dulbecco's modified Eagle's medium plus 10% foetal bovine serum, 100 U ml^−1^ penicillin, and 100 *μ*g ml^−1^ streptomycin, in 5% CO_2_ at 37°C.

### Patients and tissue samples

The expression of PPAR*δ* was examined by immunohistochemistry in the following set of colorectal samples: normal mucosa (*n*=32), adenomatous polyps (*n*=23), and various stages of carcinomas (*n*=32). Tissue samples were consecutively collected in the years 2002–2003 during surgery or during endoscopic polypectomy at the Department of Surgery, Osaka University. None of the patients had a history of family syndromes for CRC. The samples of normal mucosa were cut in the longitudinal direction, and the polyps and carcinomas were cut across the maximum diameter. These samples were fixed in buffered formalin at 4°C overnight, processed through graded ethanol solutions, and embedded in paraffin. The resected samples were used with the approval of the ethical committee of Osaka University. Adenomatous polyps were 16 tubular and seven tubulovillous adenoma.

There were 23 male and nine female CRC patients, with a mean age of 59.3 years (range, 42–80 years) at surgery. Primary tumours were distributed in the colon (*n*=14) and rectum (*n*=18). The tumours were well-differentiated adenocarcinomas (*n*=11), moderately differentiated adenocarcinomas (*n*=20), and poorly differentiated adenocarcinoma (*n*=1). Eleven patients had lymph node metastasis and 21 patients were node-negative. Dukes' staging classified nine patients as stage A, 10 patients as stage B, nine patients as stage C, and four patients as stage D.

### Antibodies

Rabbit anti-human PPAR*δ* polyclonal antibody (sc-7197, H-74) was obtained from Santa Cruz Biotechnology (Santa Cruz, CA, USA). This antibody recognises amino acids 2–75 mapping at the amino-terminus of PPAR*δ* of human origin and crossreacts with mouse and rat PPAR*δ*. Mouse anti-human *β*-catenin monoclonal antibody was purchased from Transduction Laboratories (Lexington, KY, USA). The rabbit anti-human Ki-67 polyclonal antibody was purchased from DAKO (Carpinteria, CA, USA). The rabbit anti-human actin antibody was purchased from Sigma-Aldrich (St Louis, MO, USA).

### Haematoxylin and eosin staining and immunohistochemistry

Tissue sections (4 *μ*m thick) were deparaffinised in xylene, rehydrated, and stained with haematoxylin and eosin (H&E) solution. The specimens were histologically diagnosed by two pathologists from the Department of Pathology, Osaka University. For immunostaining, sections were mounted on charged glass slides, boiled for antigen retrieval, and then processed for immunohistochemistry, as described previously (Takemasa *et al*, 2000; Yamamoto *et al*, 2003), using the Vectastain ABC peroxidase kit (Vector Laboratories, Burlingame, CA, USA). In the primary antibody reaction, the slides were incubated with appropriate antibodies for 1 h at room temperature. The dilution of each antibody was 1:40 for PPAR*δ* antibody, 1:1000 for *β*-catenin antibody, and 1:50 for Ki-67 antibody. For negative control, nonimmunised rabbit or mouse IgG (Vector Laboratories) or PBS alone was used as a substitute for the primary antibody to exclude possible false-positive responses from secondary antibody binding or from nonspecific binding of IgG. The entire series of samples was stained twice using separately prepared sections, and no discrepant staining results were noted.

### Immunohistochemical assessment

All immunostained tissue sections were evaluated by two investigators (TO and HY). Samples were coded without indicating the clinical and pathological background of the patients. In each section, 10 high-power fields were selected, and a total of at least 1000 cells were evaluated. The cell populations exhibiting an association between PPAR*δ* expression and malignant morphology were assessed in the same manner. The results of cytoplasmic staining were expressed as a percentage of positive cells, and the intensity of staining was estimated on a scale from 0 to 3 (negative, weak, moderate, and strong). The total score was determined by multiplication of the percentage of positive cells and staining intensity, ranging from 0 to 300, as reported previously (Krajewska *et al*, 1996; Shamma *et al*, 2000). For the assessment of nuclear expression, the percentage of positive cells was examined because staining intensity was routine.

### Transduction of PPAR*δ* complementary DNA (cDNA)

The mammalian expression vector pCMX-mPPAR*δ*, encoding mouse PPAR*δ* cDNA (length 1.3 kb) was a generous gift from Professor Ronald M Evans (Salk Institute, San Diego, CA, USA). A pcDNA3 vector encoding a neomycin-resistant sequence was purchased from Invitrogen (Carlsbad, CA, USA). Co-transfection was carried out with pcDNA3 and PPAR*δ* plasmid or pCMX vector at 0.5 and 2 *μ*g, respectively, into intestinal IEC18 cells using Lipofectin Reagent (Life Technologies Inc. Gaithersburg, MD, USA). Eight hours after transfection, cells were transferred from a 60-mm dish into a 150-mm dish and selected for 10 days in the presence of 0.9 mg ml^−1^ of G418 (Life Technologies).

### Western blot analysis

Western blot analysis was performed as described previously (Yamamoto *et al*, 1999). Briefly, the protein samples (50 *μg*) were separated using 10% polyacrylamide gel electrophoresis, followed by electroblotting onto a polyvinylidene difluoride membrane. The membrane was incubated with the primary antibodies at the appropriate concentrations (1 *μ*g ml^−1^ for PPAR*δ* antibody, 1:1000 for actin) for 1 h. Protein bands were detected using the Amersham ECL detection system (Amersham Biosciences Corp., Piscataway, NJ, USA).

### Quantitative real-time PCR for PPAR*δ* mRNA

Total cellular RNA was extracted using TRIZOL reagent (Life Technologies Inc., Gaithersburg, MD, USA). Complementary DNA was generated from 1 *μ*g RNA with avian myeloblastosis virus reverse transcriptase (Promega, Madison, WI, USA). Quantitative real-time PCR was performed using LightCycler™ (Idaho Technology Inc., Salt Lake City, Utah, USA), as described previously (Yamamoto *et al*, 2003). Quantification data from each sample were analysed using LightCycler™ analysis software. The transcription value of PPAR*δ* was determined by plotting on a standard curve constructed using HCT116 colon cancer cells. The amount of each transcript was normalised according to that of *β*-actin housekeeping gene quantified with the same sample. The primer sequences were as follows: *β*-actin sense, 5′-GAAAATCTGGCACCACACCTT-3′; *β*-actin antisense, 5′-GTTGAAGGTAGTTTCGTGGAT-3′; PPAR*δ* sense: 5′-GTGGACCTGTCACTGTCTTGTAC-3′; and PPAR*δ* antisense: 5′-CTTCCTCTTGGAGAAGATCAGC-3′.

### Statistical analysis

Statistical analysis was performed using the StatView J-5.0. program (Abacus Concepts Inc., Berkeley, CA, USA). Associations between the discrete variables were assessed using Fisher's exact tests. Data were reported as mean±s.d., and mean values were compared using the Mann–Whitney test. *P*-values <0.05 were accepted as statistically significant.

## RESULTS

### Validation of specificity of the anti-PPAR*δ* antibody

**Figure 1:**
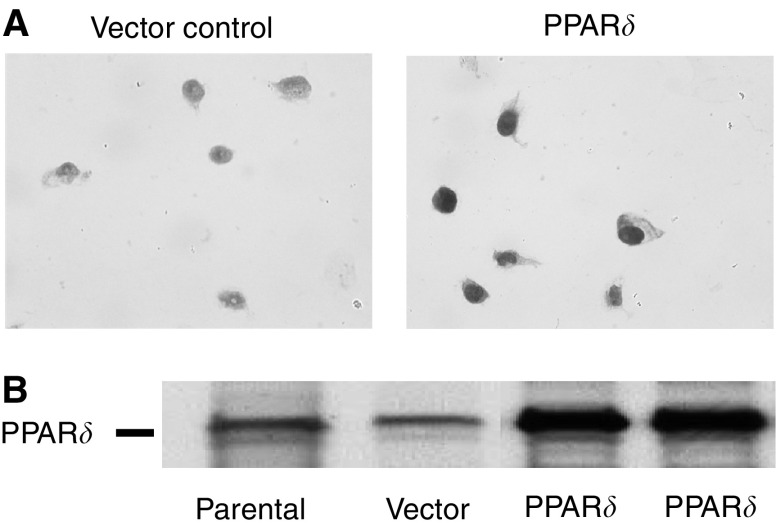
Specificity of anti-PPAR*δ* antibody. (**A**) Immunocytochemistry with anti-PPAR*δ* antibody. After selection with G418 (0.9 mg ml^−1^), pooled cultures from each dish were stained with anti-PPAR*δ* antibody. Peroxisome proliferator-activated receptor *δ*-transfected cultures of IEC18 intestinal cells displayed intense PPAR*δ* staining in comparison to the weak PPAR*δ* staining noted in the control cultures. (**B**) Western blotting using anti-PPAR*δ* antibody showed that PPAR*δ*-introduced cultures displayed prominent bands for the PPAR*δ* protein compared with parental and vector control cells.

Immunocytochemistry showed that PPAR*δ*-transfected cultures displayed intense PPAR*δ* staining in comparison to the weak PPAR*δ* staining noted in the control cultures (Figure 1A). Western blotting using anti-PPAR*δ* antibody showed that PPAR*δ*-introduced cultures displayed prominent bands for the PPAR*δ* protein compared with parental and vector control cells (Figure 1B). These results indicate that PPAR*δ* antibody specifically reacts with the PPAR*δ* protein.

### PPAR*δ* expression in CRC tissues

**Figure 2:**
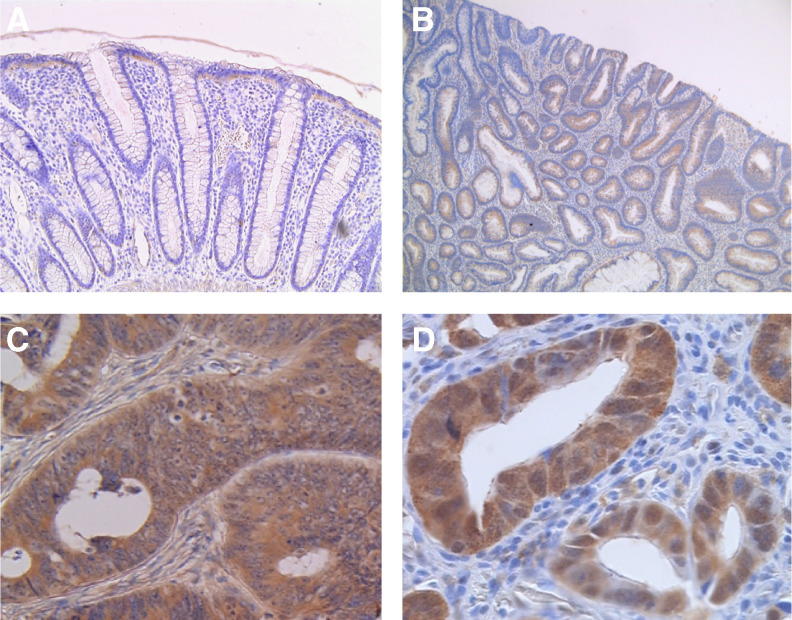
Immunohistochemistry for PPAR*δ*. (**A**) In normal colonic mucosa, the PPAR*δ* protein was weakly detected in the epithelial cells on the luminal surface of the mucosal glands. (**B**) In adenomatous polyps, PPAR*δ* was weakly expressed in the cytoplasm. (**C**) Cytoplasmic expression and (**D**) nuclear expression in carcinoma tissues. Magnifications: **A**: × 50; **B**: × 20; **C**: × 100; **D**: × 150.

In normal colonic mucosa, PPAR*δ* protein was detected in the epithelial cells on the luminal surface of the mucosal glands (Figure 2A). In adenomatous polyps, PPAR*δ* was weakly expressed in eight of 23 samples (34.8%) in the cytoplasm (Figure 2B). On the other hand, all the carcinoma tissues expressed the PPAR*δ* protein to various extents, in the cytoplasm and/or nucleus (Figures 2C and D). More than half of the cancer tissues exhibited nuclear expression at less than 10%, whereas a considerable number of the cancer tissues showed cytoplasmic PPAR*δ* expression (Table 1). Peroxisome proliferator-activated receptor*δ* expression in adenomatous polyps and cancer tissues with regard to their localisation and expression extent is summarised in Table 1. The differences in cytoplasmic PPAR*δ* expression between adenomatous polyps and cancers were significant (*P*<0.0001).

The cancer specimens were divided into two groups (high expression: *n*=15 (46.9%) and low expression: *n*=17 (53.1%)) based on the mean value of a cytoplasmic PPAR*δ* score of 160 (see Materials and Methods regarding score determination). Comparison of these two groups showed no differences in the various clinical and pathological parameters listed in Table 2. In addition, nuclear expression level (mean value 8.3% at the cutoff point) did not correlate with clinical and pathological parameters in these two groups (data not shown).

### Western blot analysis

**Figure 3:**
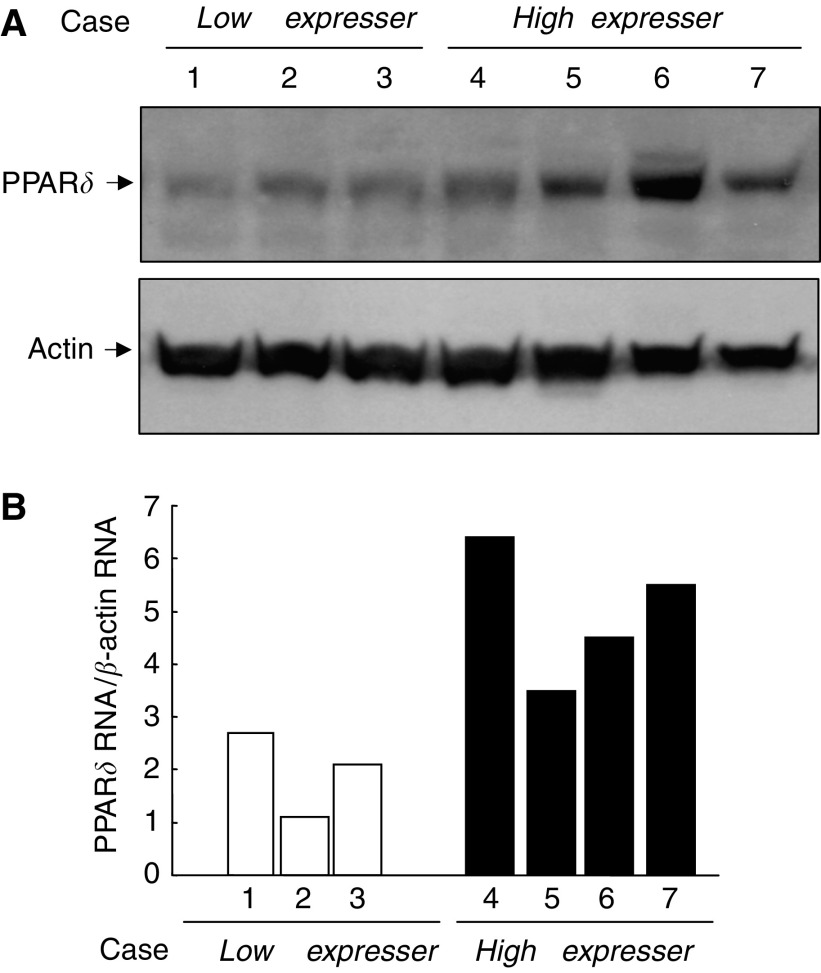
(**A**) Western blot analysis for the PPAR*δ* protein. Protein levels were quantified for three CRC cases with low cytoplasmic PPAR*δ* and four CRC cases with high cytoplasmic PPAR*δ* levels. Peroxisome proliferator-activated receptor *δ* levels in cancer tissues correlated well with those detected by immunohistochemistry. (**B**) Quantitative RT–PCR for PPAR*δ* mRNA. The same tissue samples used in Western blot analysis were subjected to quantitative RT–PCR for PPAR*δ* mRNA quantification. Samples exhibiting high levels of PPAR*δ* protein expression generally exhibited high levels of PPAR*δ* mRNA, whereas those with low PPAR*δ* protein levels exhibited low levels of PPAR*δ* mRNA.

Among the above series, three CRC cases with low cytoplasmic PPAR*δ* and four CRC cases with high cytoplasmic PPAR*δ* were subjected to Western blot analysis to determine PPAR*δ* protein levels (Figure 3A). Colorectal cancer samples expressed various levels of the PPAR*δ* protein that correlated well with those detected by immunohistochemistry.

### Level of PPAR*δ* mRNA

The same tissue samples used in Western blot analysis were subjected to quantitative RT–PCR for PPAR*δ* mRNA quantification. Samples exhibiting high expression of PPAR*δ* protein generally also exhibited high levels of PPAR*δ* mRNA, whereas those with low PPAR*δ* protein levels exhibited low levels of PPAR*δ* mRNA (Figure 3B).

### Relationship between PPAR*δ* expression and Ki-67 expression

**Figure 4:**
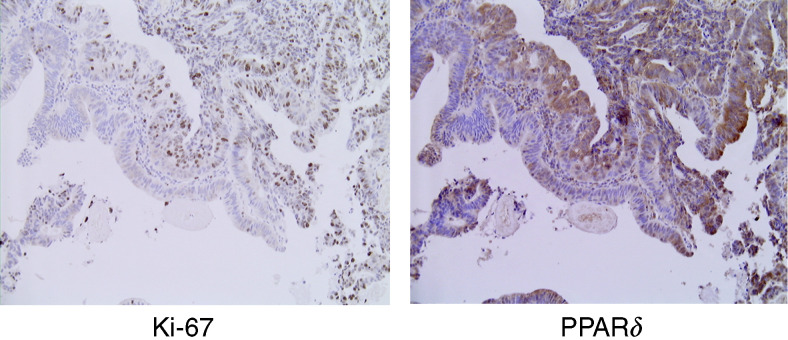
Comparative immunohistochemistry for PPAR*δ* and Ki-67 expression. A concordant distribution of Ki-67-expressing colon cancer cells and those with cytoplasmic accumulation of PPAR*δ*. Magnifications: × 20.

To investigate the possible involvement of PPAR*δ* in cell growth, we compared the expression of PPAR*δ* and Ki-67, a cell proliferation marker, in the serial sections. Nuclear Ki-67-positive cells were localised at the proliferative zone of the normal epithelium, but were randomly distributed in cancer tissue with Ki-67 indices ranging from 22.1 to 80.0% (mean value: 49.6±15.1%). Several CRC samples displayed a concordant distribution of Ki-67-expressing cells and cells with cytoplasmic accumulation of PPAR*δ* (Figure 4), but not nuclear PPAR*δ* (data not shown). Analysis of all the samples, however, found no significant correlation between the cytoplasmic expression of PPAR*δ* and Ki-67 (data not shown).

### PPAR*δ* expression and malignant morphology of CRC cells

**Figure 5:**
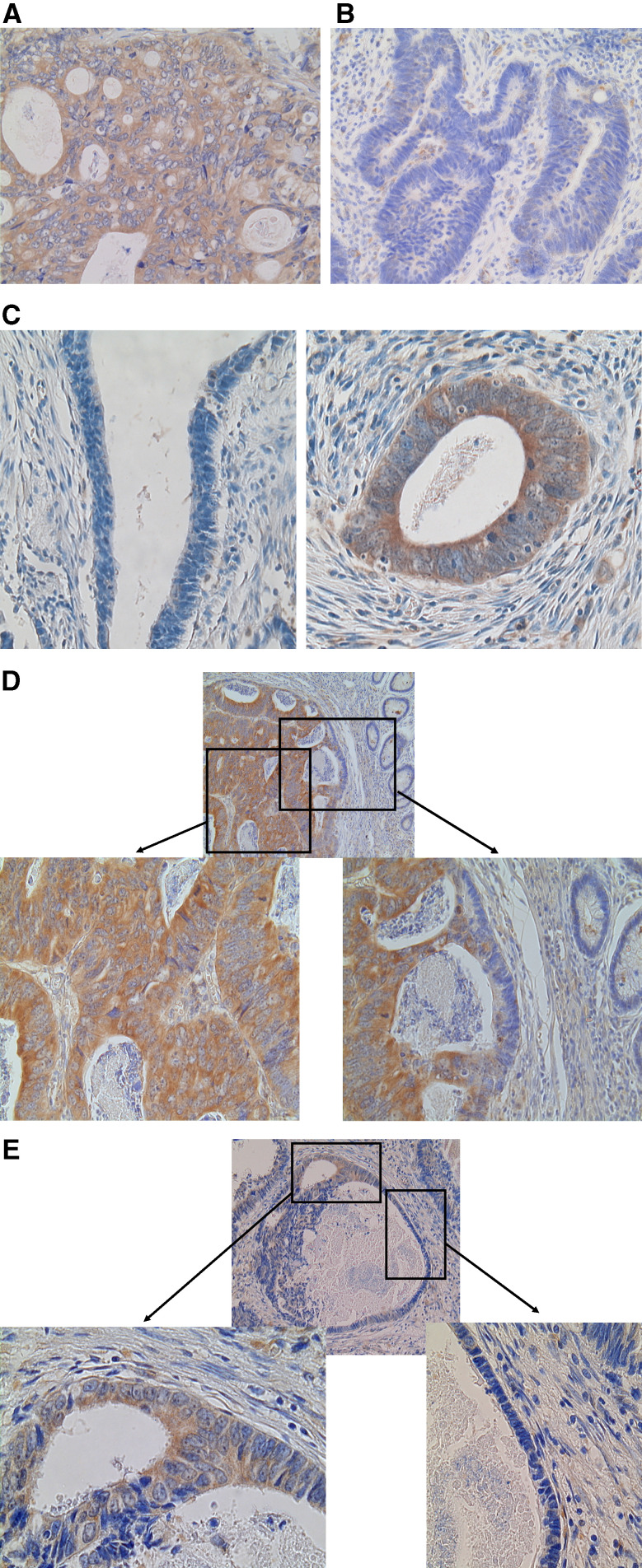
Peroxisome proliferator-activated receptor *δ* expression and morphology of CRC cells. (**A**) PPAR*δ*-positive cancer cells presented morphological features associated with high malignant potential including a large nucleus, globular nuclear shape, appearance of a distinct nucleolus, and loss of cellular polarity. (**B**) PPAR*δ*-negative cancer cells presented a morphology (e.g., oval and small nucleus and preserved cellular polarity) associated with low malignant potential. (**C**) PPAR*δ*-negative and -positive cancer cells were present within the same CRC tissue, with maintenance of their respective specific morphological features. This pattern was maintained even when PPAR*δ*-positive and -negative cells were aligned side by side within a single cancer nest (**D**) or gland (**E**). Magnifications: **A**–**C**: × 100; **D**–**E**: upper panel × 20, lower panel × 100.

During the course of this study, we found that the cancer cells with cytoplasmic accumulation of PPAR*δ* often exhibited morphological features associated with a highly malignant phenotype. These features included a large nucleus, globular nuclear shape, appearance of distinct nucleolus, and loss of cellular polarity (Figure 5A). In contrast, PPAR*δ*-negative cancer cells had morphological features associated with a low malignant phenotype, such as an oval and small nucleus without a distinct nucleolus, and preserved cellular polarity (Figure 5B). It was of interest that a cancer tissue often contained both PPAR*δ*-positive and -negative areas, with maintenance of these respective, specific morphological features (Figure 5C). Moreover, this rule was maintained even when PPAR*δ*-positive and -negative cells were aligned next to each other within a single cancer nest or gland (Figure 5D and E). Table 3 summarises the levels of PPAR*δ* expression, nuclear size and shape index, and presence or absence of a distinct nucleolus in these samples.

Further microscopy analysis, as indicated in the Materials and Methods, revealed that this association between PPAR*δ* expression and malignant morphological features was a common rule in the majority of cancer samples tested. Thus, 100% of cancer cells followed the rule in the 10 CRCs, 50–99% of cancer cells followed the rule in 18 CRCs, and 1–49% of cells in three CRCs adhered to the pattern. One CRC sample did not exhibit this association.

## DISCUSSION

With regard to human tissue, increased expression of PPAR*δ* was first reported in a small set of CRC tumours (He *et al*, 1999; Gupta *et al*, 2000). To elucidate further the expression and role of PPAR*δ* in human colorectal tumour, we examined expression of PPAR*δ* in multistage carcinogenesis of the colorectum and found that PPAR*δ* expression increased from normal mucosa to adenomatous polyps to cancer tissues. Furthermore, we found that PPAR*δ* expression was tightly associated with highly malignant morphology of colon cancer cells. Thus, the present data suggest a pivotal role of PPAR*δ* in human CRC tissue. These findings are consistent with the recent reports that PPAR*δ* mRNA is overexpressed in more than half of CRCs (Yang *et al*, 2006) and that PPAR*δ* protein expression is elevated in adenomas in Apc^min^ mice and in colon tumours familial adenomatous polyposis patients (Knutsen *et al*, 2005). Although clinicopathological correlations were not obtained with PPAR*δ* expression in CRC, we should emphasise that the relatively small number of CRC specimens examined may have given a low statistical power.

In spite of these indications, however, whether PPAR*δ* is a good target for chemoprevention and/or treatment of CRC remains controversial. It has been reported that a polymorphism in PPAR*δ* modifies the protective effects of nonsteroidal anti-inflammatory drugs on colorectal adenomas (Siezen *et al*, 2006), but other investigators have not reached the same conclusions in the context of CRC (McGreavey *et al*, 2005). As mentioned in the Introduction, PPAR*δ* was found to be unnecessary for small intestinal polyp formation (Barak *et al*, 2002), but PPAR*δ* attenuated polyp formation in chemical and genetic models (Harman *et al*, 2004; Reed *et al*, 2004). By contrast, it has been reported that inactivation of the PPAR*δ* gene results in reduced tumorigenicity and *in vivo* growth of HCT116 colon cancer cells (Park *et al*, 2001) and that a specific PPAR*δ* agonist enhanced *in vivo* growth of intestinal adenoma of Apc^min^ mice (Gupta *et al*, 2004). Moreover, a decrease in PPAR*δ* expression by nitric-oxide-donating aspirin isomers was found to be proportional to their tumour inhibitory effects in Apc^min^ mice (Ouyang *et al*, 2006).

A recent report by Wang *et al* might be a clue to the puzzle. They reported that prostaglandin E_2_-mediated enhancement of intestinal adenoma of Apc^min^ mice was negated in Apc^min^/PPAR*δ*^*−/−*^ mice (Wang *et al*, 2004a, 2004b). Peroxisome proliferator-activated receptor*δ* may mediate the antiapoptotic effect through activation by prostacyclin (PGI(2)), a major prostaglandin with antiapoptotic activity (Gupta *et al*, 2000; Cutler *et al*, 2003). There is also cumulative evidence regarding the antiapoptotic effects of PPAR*δ* in keratinocyte and colon cancer cells (Michalik *et al*, 2001; Di-Poi *et al*, 2002; Shureiqi *et al*, 2003; Gupta *et al*, 2004). These findings suggest that PPAR*δ* may play a certain tumour-promoting role in intestinal tumours or CRC cells by modulating cell survival and apoptosis, which is in line with our observation that PPAR*δ* was exclusively expressed in those CRC cells that also exhibited highly malignant morphology.

Cellular atypia is the pathological hallmark for estimating the malignant potential of lesions. Studies with large numbers of CRC patients (*N*=343, 100, 90, and 64, respectively) (Ambros *et al*, 1990; Mitmaker *et al*, 1991; Fernandez-Lopez *et al*, 1999; Ikeguchi *et al*, 1999) have shown that the nuclear area, the large maximum nucleus diameter, or nuclear shape index, when determined with the aid of nuclear morphometry, is associated with cancer metastasis or poor prognosis. During the current study, we found that PPAR*δ*-expressing cancer cells often presented such nuclear features, whereas PPAR*δ*-negative cells did not. Surprisingly, this association was found in the majority of CRC cases and was maintained even when PPAR*δ*-positive cells and PPAR*δ*-negative cells were positioned next to each other. These findings indicate that cytoplasmic accumulation of PPAR*δ* could be a sensitive marker of CRC cells with the potential for high malignancy. Recently, Hinoi *et al* showed that loss of CDX2 was a marker for large-cell minimally differentiated carcinomas of the colon (Hinoi *et al*, 2001), and we are not aware of other molecular markers tightly associated with CRC cell morphology.

PPAR*δ* is known as a nuclear receptor, and we indeed found that introduction of PPAR*δ* cDNA resulted in nuclear expression in IEC18 cells, whereas immunohistochemistry showed cytoplasmic accumulation of PPAR*δ* in CRC tissues. We believe that the latter findings do not represent nonspecific binding of the PPAR*δ* antibody in tissue samples; *β*-catenin staining in the same tumour series indicated that cytoplasmic PPAR*δ* was selectively induced in CRC cells, possibly as a result of aberrant accumulation of oncogenic *β*-catenin (data not shown, our unpublished data). Therefore, it is postulated that cytoplasmic accumulation of PPAR*δ* may be necessary for the proteins to be available for their nuclear role whenever required. It is also possible that nuclear PPAR*δ* might be present at a low level but was not detectable because of the limited sensitivity of the immunohistochemical analysis.

Our immunohistochemical study of Ki-67 and PPAR*δ* did not identify positive effects of PPAR*δ* on *in vivo* cell growth. We found by *in vitro* assay that the growth of intestinal cells was stimulated by the introduction of PPAR*δ* cDNA (data not shown, our unpublished data), findings that are consistent with other reports that PPAR*δ* enhances the *in vitro* growth of breast and prostate cancer cells (Stephen *et al*, 2004). These results suggest that other positive and negative regulators could be simultaneously exerting their effects on cell growth.

In conclusion, the present study using CRC tissue samples showed that PPAR*δ* expression increased during multistage carcinogenesis. Our data suggest that the association of PPAR*δ* with CRC malignant cellular morphology suggests a pivotal role for PPAR*δ* in these cells.

## Figures and Tables

**Table 1:** PPAR*δ* expression in colorectal tumour

*Nuclear expression* a			
% Positive	0–10	10<*X*<30	30–100
Cancer	20 (62.5%)	10 (31.3%)	2 (6.2%)
			
*Cytoplasmic expression*			
PPAR score^b^	0–50	50<*X* <150	150–300
Adenoma	18 (78.3%)	5 (21.7%)	0 (0%)
Cancer	3 (9.4%)	13 (40.6%)	16 (50.0%)

PPAR=peroxisome proliferator-activated receptor.

a Adenomatous polyp showed no nuclear expression.

b Determined by multiplication of staining intensity (0–3) and positivity (0–100).

**Table 2:** PPAR*δ* and clinicopathological characteristics

		**PPAR*δ***		
**Clinicopathological characteristic**	***n***	**High**	**Low**	***P*-value**
Age (year)^a^	32	60.8±8.3	58.1±8.6	0.386
Tumour size (cm)^a^	32	4.2±1.3	4.7±2.0	0.467
				
*Gender*				
Male	23	12	11	0.337
Female	9	3	6	
				
*Tumour site*				
Colon	14	7	7	0.755
Rectum	18	8	10	
				
*Degree of differentiation*				
Well	11	6	5	0.519
Mod/por^b^	21	9	12	
				
*Depth of invasion*				
∼mp	10	6	4	0.316
ss∼	22	9	13	
				
*Lymph node metastasis*				
Absent	21	10	11	0.907
Present	11	5	6	
				
*Stage*				
Dukes A, B	19	10	9	0.430
C, D	13	5	8	
				
Total	32	15	17	

Mod=moderately differentiated adenocarcinoma; mp=muscularis propria; PPAR*δ*=peroxisome proliferator-activated receptor *δ*; por=poorly differentiated carcinoma; ss=subserosa; well=well-differentiated adenocarcinoma.

a Data are mean±s.d.

b Por tumour was only one that had a low PPAR*δ* expression.

**Table 3:** PPAR*δ* expression and cancer cell morphology

**Case**	**Cell type**	**Intensity of PPAR*δ* staining**	**Nuclear size index^a^**	**Nuclear shape index^b^**	**Presence of distinct nucleolus**
A	PPAR (+)	77.0	170.4	0.68	Yes
B	PPAR (−)	3.8	77.0	0.55	No
C	PPAR (+)	41.3	239.7	0.64	Yes
	PPAR (−)	8.0	80.6	0.50	No
D	PPAR (+)	45.7	80.1	0.78	Yes
	PPAR (−)	4.0	30.4	0.59	No
E	PPAR (+)	29.6	275.1	0.72	Yes
	PPAR (−)	2.0	50.0	0.54	No

PPAR=peroxisome proliferator-activated receptor.

a Major axis multiplied by minor axis.

b Defined as the degree of circularity of the nucleus; minor axis divided by major axis. A perfect circle was recorded as 1.0.
